# Does Contract Practice Pay?

**Published:** 1902-10-11

**Authors:** 


### DOES CONTRACT PRACTICE PAY?

Much discussion has taken place from time to time as to whether contract practice pays. That it does not pay under the conditions imposed upon it by the medical aid associations, and that the operation of these associations has had a doubly injurious effect upon professional remuneration in that in addition to cutting down the contract payment to the lowest conceivable limit, they have also intruded into those classes from among whom private practice is drawn is clear enough. But whether contract practice when organised by the medical men themselves, for their own benefit, under their own control, and with full guarantees against encroachment upon private practice, would or would not pay is another matter. It is admittedly difficult to obtain any satisfactory information as to the amount of attendance which a club doctor is called upon to give in return for the subscriptions of the club members. Yet it is upon this that the whole question hinges. Hence much interest attaches to a paper by Dr. Alfred Cox,[1] in which he gives details as to the working of two friendly society lodges with which he has been connected for the past three and a half years. One belongs to the Ashton Unity of Shepherds and contains 150 members; the other to the Order of Druids and numbers at present 75. The Shepherds pay at the rate of 3s. per head per annum, the Druids at first paid 3s., but now pay 3s. 6d., per head per annum. The members are almost exclusively genuine working men, and in his district the question of abuse of the medical benefits by well-to-do men has rarely arisen. The membership consists of just the same kind of men as, in private practice in his district, pay the standard fee of 2s. 6d. per attendance, being mostly men in receipt of wages varying from 25s. to 38s. per week. Now, after making careful note of every visit and consultation, except where the member has merely called to have his sick-sheet signed, and entering all examinations for entrance as attendances the result shows that the Shepherds pay on an average 1s. 2¼d. perattendance, and the Druids 1s. 5½d. To put the two sets of figures together, and strike an average for the three and a half years, Dr. Cox has received in that time £116 7s. 9d., for which sum he has made 1,708 attendances for an average of 1s. 3½d. per attendance. Now comes a calculation with which some people will not perhaps agree. Dr. Cox says that he has made a calculation of the out-of-pocket cost of each attendance made in his practice. This he has done by deducting from his income everything but the money used in making that income. "For example," he says, "I deduct all money spent in cost of living, any savings, anything spent on pleasure, on luxuries generally, on clothes, etc. The remainder will be the cost of carrying on the practice. In this way I estimate that 1s. will be the cost of each attendance in my practice. This is making no charge for one's time and skill. Now, after allowance for bad debts, I find that the average sum paid by my private patients, who are all working-class people, is about 2s. 3d. per attendance." From this he concludes that he gets 1s. less for a Friendly Society attendance than for one on an ordinary private patient, the only difference in the service rendered being that the club patient brings his own bottle and comes more frequently to the house in preference to sending for a visit than does the private patient. Putting the matter thus, he comes to this conclusion: "From Jones who lives in X Street I get 1s. 3d. for my time and skill, whereas from Brown who lives next door, and is just as well off, but who is a member of a Friendly Society, I get only 3½d." Now, we are not quite sure that this is a fair way of calculating the matter, but that we must leave to our readers; the point of interest seems to be that as a result of a careful painstaking inquiry, Dr. Cox arrives at the result that he receives on an average 1s. 3½d. for his club attendances, including both his visits and the consultations at his own house, and this from a club subscription which is much lower than the penny a week about which some people speak in such slighting terms. Truly it is not much, but we fancy it is more than a good many Poor-Law appointments would work out to—appointments as to the acceptance of which not a word is said. It is clear that for the decision of this question the first thing we want is a series of facts similar to those collected by Dr. Alfred Cox. Having these, each man must decide for himself whether contract practice pays or not.

[1] Brit. Med. Jour., Oct. 4.

